# Dairy-Derived and Egg White Proteins in Enhancing Immune System Against COVID-19

**DOI:** 10.3389/fnut.2021.629440

**Published:** 2021-07-12

**Authors:** Gaber El-Saber Batiha, Mohammed Alqarni, Dina A. B. Awad, Abdelazeem M. Algammal, Richard Nyamota, Mir I. I. Wahed, Muhammad Ajmal Shah, Mohammad N. Amin, Babatunde O. Adetuyi, Helal F. Hetta, Natália Cruz-Martins, Niranjan Koirala, Arabinda Ghosh, Javier Echeverría, Jorge Pamplona Pagnossa, Jean-Marc Sabatier

**Affiliations:** ^1^Department of Pharmacology and Therapeutics, Faculty of Veterinary Medicine, Damanhour University, Damanhour, Egypt; ^2^Department of Pharmaceutical Chemistry, College of Pharmacy, Taif University, Taif, Saudi Arabia; ^3^Department of Food Hygiene, Faculty of Veterinary Medicine, Benha University, Benha, Egypt; ^4^Department of Bacteriology, Immunology, and Mycology, Faculty of Veterinary Medicine, Suez Canal University, Ismailia, Egypt; ^5^Department of Biochemistry and Molecular Biology, Faculty of Science, Egerton University, Njoro, Kenya; ^6^Department of Pharmacy, University of Rajshahi, Rajshahi, Bangladesh; ^7^Department of Pharmacognosy, Faculty of Pharmaceutical Sciences, Government College University, Faisalabad, Pakistan; ^8^Department of Pharmacy, Atish Dipankar University of Science and Technology, Dhaka, Bangladesh; ^9^Pratyasha Health Biomedical Research Center, Dhaka, Bangladesh; ^10^Department of Natural Sciences, Faculty of Pure and Applied Sciences, Precious Cornerstone University, Ibadan, Nigeria; ^11^Department of Medical Microbiology and Immunology, Faculty of Medicine, Assiut University, Asyut, Egypt; ^12^Faculty of Medicine, University of Porto, Porto, Portugal; ^13^Institute for Research and Innovation in Health (i3S), University of Porto, Porto, Portugal; ^14^Laboratory of Neuropsychophysiology, Faculty of Psychology and Education Sciences, University of Porto, Porto, Portugal; ^15^Department of Natural Products Research, Dr. Koirala Research Institute for Biotechnology and Biodiversity, Kathmandu, Nepal; ^16^Laboratory of Biotechnology, Faculty of Science and Technology, University of Macau, Taipa, Macau; ^17^Microbiology Division, Department of Botany, Gauhati University, Guwahati, India; ^18^Departamento de Ciencias del Ambiente, Facultad de Química y Biología, Universidad de Santiago de Chile, Santiago, Chile; ^19^Biological Sciences Department, Federal University of Lavras (UFLA), Lavras, Brazil; ^20^Université Aix-Marseille, Institut de Neuro-Physiopathologie (INP), UMR 7051, Faculté de Pharmacie, Marseille, France

**Keywords:** SARS-CoV-2, coronavirus disease 2019, immunomodulation, nutritional therapy, nutrients

## Abstract

Coronavirus disease (COVID-19) is a global health challenge, caused by the severe acute respiratory syndrome coronavirus-2 (SARS-CoV-2) triggers a plethora of respiratory disturbances and even multiple organs failure that can be fatal. Nutritional intervention is one of the key components toward to a proper management of COVID-19 patients, especially in those requiring medication, and should thus be considered the first-line treatment. Immuno-modulation and -stimulation are currently being explored in COVID-19 management and are gaining interest by food and pharmaceutical industries. Various dietary combinations, bioactive components, nutrients and fortified foods have been reported to modulate inflammation during disease progression. Dietary combinations of dairy-derived products and eggs are gaining an increasing attention given the huge immunomodulatory and anti-inflammatory properties attributed to some of their chemical constituents. Eggs are complex dietary components containing many essential nutrients and bioactive compounds as well as a high-quality proteins. Similarly, yogurts can replenish beneficial bacteria and contains macronutrients capable of stimulating immunity by enhancing cell immunity, reducing oxidative stress, neutralizing inflammation and regulating the intestinal barriers and gut microbiome. Thus, this review highlights the impact of nutritional intervention on COVID-19 management, focusing on the immunomodulatory and inflammatory effects of immune-enhancing nutrients.

## Introduction

Coronavirus disease (COVID-19) triggered by the severe acute respiratory syndrome-coronavirus-2 (SARS-CoV-2) led to multiple respiratory disturbances, multiple organs failure and even death, ultimately representing a global health challenge. The virus, first reported in December 2019, in the Wuhan city - China (1), has spread worldwide and has been classified by the World Health Organization (WHO) as a global pandemic (2, 3). Clinical presentations by most COVID-19 patients requiring admission include severe inflammation, respiratory failure and reduced appetite (4).

**Figure 1:**
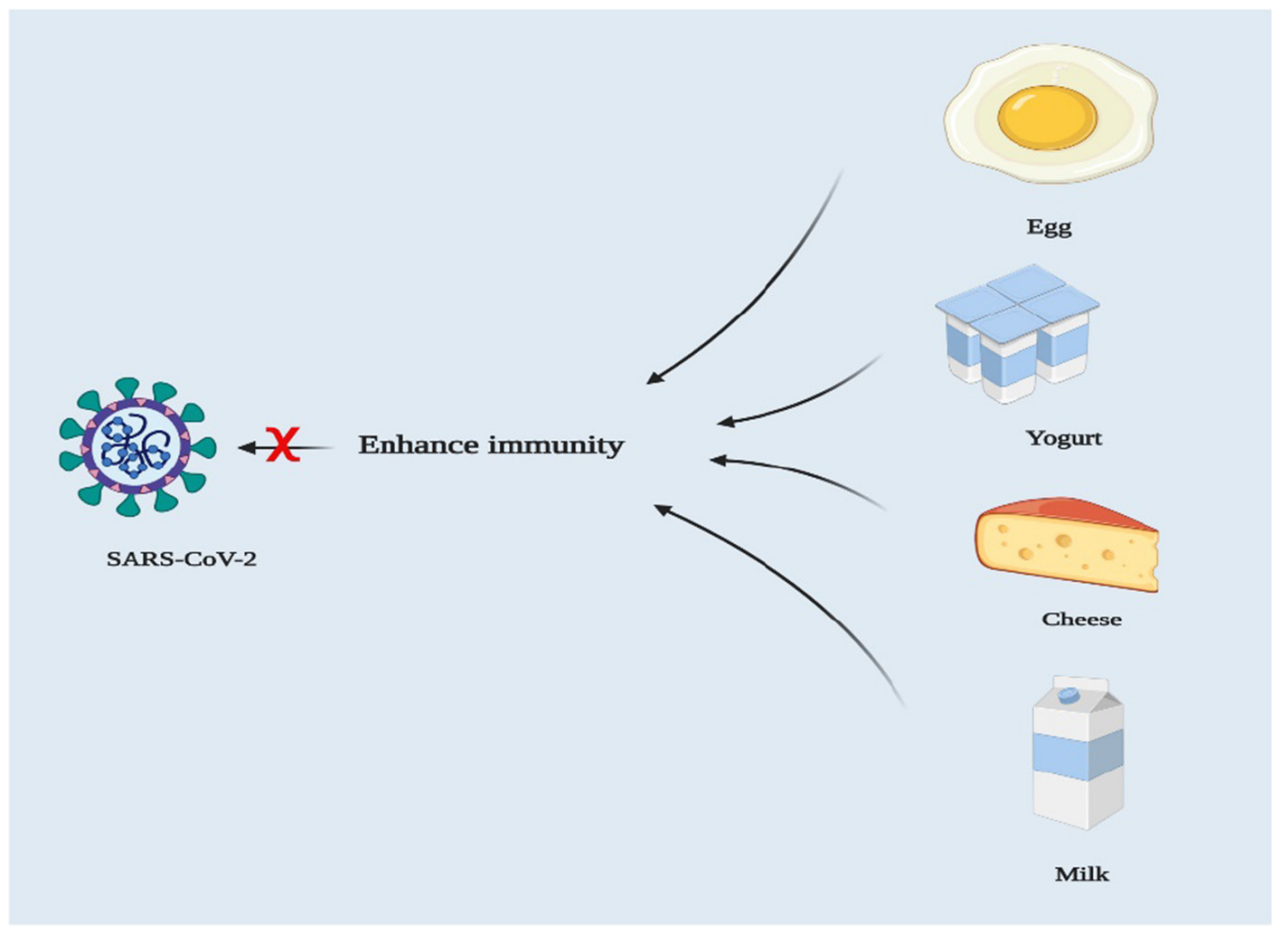
Food sources enhancing the immune response.

Nutritional intervention, also regarded as the first-line treatment, is among the core components involved in comprehensive management of COVID-19 patients under medication. Available clinical evidence reveals that although people of all ages are susceptible to infection, a poor prognosis and higher mortality rate has been reported in malnourished elderly individuals, immunocompromised patients and even in those with chronic diseases (5). In this sense, not only does good nutrition enhance body immunity against diseases, including COVID-19, but also shortens the recovery period (6). The elderly are especially susceptible to infection, owing to their body's declining physiological and immunological efficiency. This increases the likelihood of infections, being severe and refractory, thereby presenting an immunity challenge to the elderly who happens to contract an infectious pathogen. This elucidates the importance of systematic nutritional derangements in COVID-19 patients since immunity is weakened by inadequate nutrition (7, 8). Currently, immune modulation and stimulation is being increasingly explored for COVID-19 management and has gained interest by both food and pharmaceutical industries. In fact, the immune response is crucial for maintaining healthy human physiology by detecting and eliminating pathogens, aging, or cancer cells (9). Briefly, immunomodulation refers to the immune system ability to regulate fatal illnesses, such as acquired immunodeficiency syndrome caused by human immunodeficiency virus (HIV) (10, 11). Currently used therapeutic immunomodulators include penicillamine, cyclosporine A, cyclophosphamide, pidotimod, levamisole, thiocarbamate, imiquimod, prostaglandin, tilorone, and niridazole (12–16). However, these drugs are usually associated with undesired side effects. In addition, since no effective drug is currently available for COVID-19 treatment or adequate early phase vaccine supply for the entire populace, nutritional enhancement of immune system is the best immediate intervention for preventing SARS-CoV-2 infection. In this sense, this review presents different food sources and nutritional supplements that enhance immune function, such as egg derived proteins, milk and fermented dairy products containing different probiotics (Figure 1), that have been shown to play a key role in preventing COVID-19 and in the management of hospitalized patients with mild clinical presentations.

## Immune System Activity: A Synopsis

The primary host's defense from pathogens and harmful toxins emanates from the immune system (17). Two categories of the immune system include the adaptive and innate subsystems. The innate immune subsystem also referred to as native/natural immunity is non-specific and forms the layer of protection, usually through mechanical barriers of entry, such as the skin and mucosal tissues (17). Other forms of native immunity involve the non-specific inflammatory components, such as interferons, defensins, and cytokines, and also incorporate the bone marrow components, such as the basophils, monocytes, dendritic cells, macrophages, eosinophils, and neutrophils. As critical first-line of defense, neutrophils and macrophages are also of pivotal important in phagocytosis, the mechanisms through which macrophages recognize and neutralize pathogens and defective/cancer cells (18). Macrophages act also as immune regulators by producing different cytokines (i.e., interleukins, interferon gamma, tumor necrosis factors-α) (19). On the other side, an immunomodulator can be stratified as immunosuppressant, immunostimulant, or immunoadjuvant. In this perspective, immunotherapy can be defined as the host's immunity modulation with the desired outcome of disease treatment or management (20).

**Figure 2:**
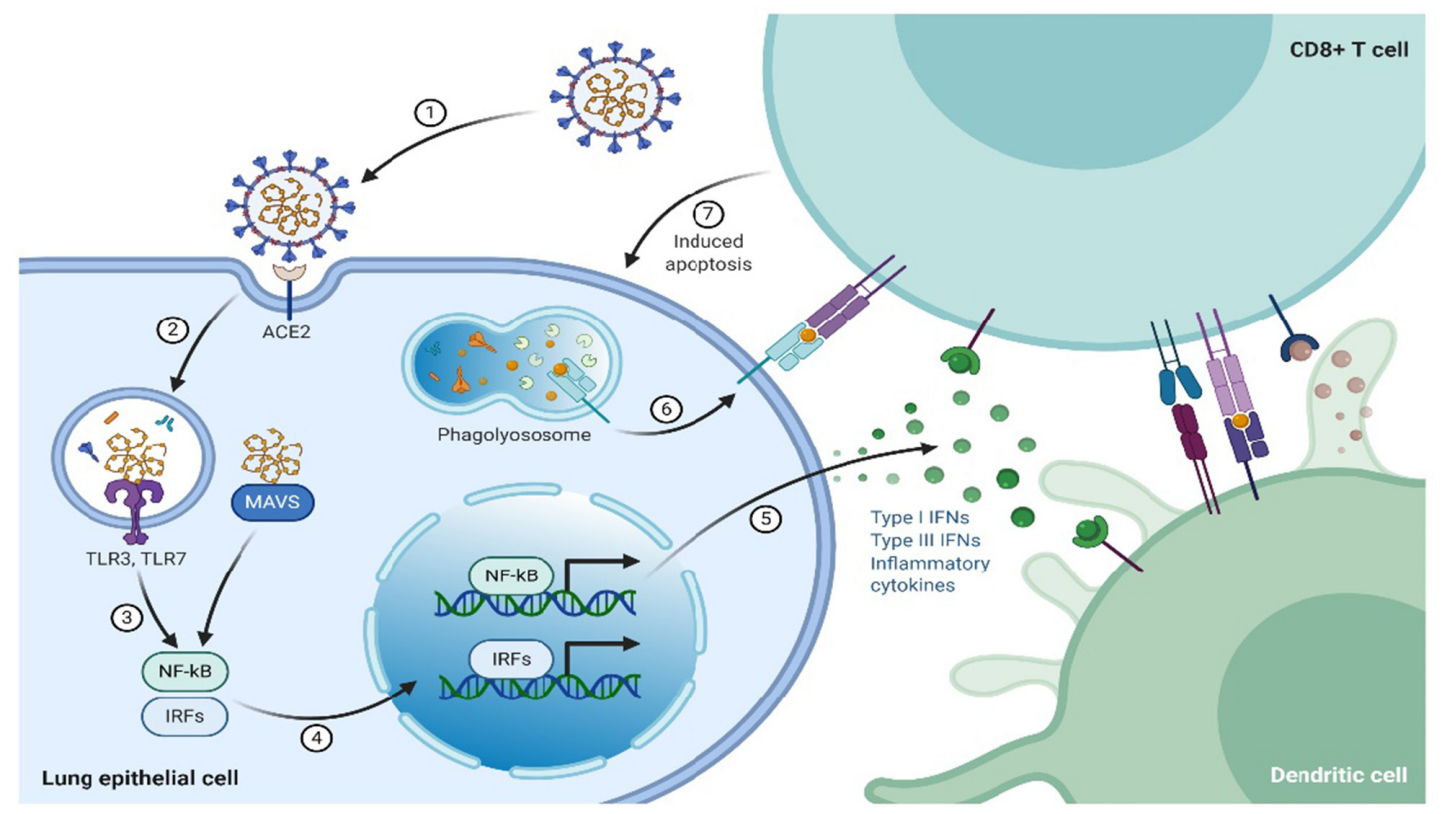
The immune system response to SARS-CoV-2.

In contrast to innate immunity, the specific response by the immune system is referred to as adaptive/acquired/specific. This kind of immune response is usually the second-line of defense and involves B and T lymphocytes (20). Adaptive immunity can either be cell-mediated or humoral immune response, where humoral immunity is mediated by B-lymphocytes through antibodies production, that are specifically directed toward the pathogen, whereas cell-mediated immune responses are initiated by T lymphocytes complexing with infected cells or pathogens, which leads to lysis of the “unwanted” cells and cytokines release, whose primary role is immune regulation. T lymphocytes/cells fall into three subsets, termed as helper (TH), cytotoxic, and suppressor or regulatory T cells. The expression of cluster of differentiation (CD) 8+ cell surface receptors characterizes the cytotoxic lymphocytes. The CD8 surface receptors play a role in recognition of endogenous antigens presented by the major histocompatibility complex class I (MHC-I) from virus-infected cells that have been lysed (Figure 2). In contrast, helper T cells, express CD4+surface receptors that recognize exogenous antigens presented by MHC-II. The reaction mechanism of TH cells entails T and B lymphocytes activation by secreting cytokines that also activate other immune cells (21, 22), whereas the suppressor T cells regulate the immune response by smothering autoimmunity, thereby maintain the self-tolerance.

## Egg as an Immune-Enhancing Food

It has been reported that various dietary combinations, bioactive components, nutrients, and fortified foods modulate inflammation during disease progression (23–25). Eggs represent a complex and controversial dietary component (26) that contains numerous essential nutrients and bioactive compounds, besides being high-quality protein source leading to divergent opinions in dietary recommendation across populations (27, 28).

### Egg Structure

Structurally, eggs are composed of albumin (63%), eggshell (9.5%), and yolk (27.5%) (29). Biochemically, they comprise of 75% water, 12% proteins, 12% lipids, various minerals and carbohydrates (30, 31). Though proteins are distributed across the different egg parts, they are mainly contained in the yolk and egg white, while small proportions occur in the eggshell and shell membrane (32, 33). Lipids exclusively occur in the egg yolk mainly as lipoproteins (30, 32), while the bulk of minerals is found in the eggshell. As minor egg components, carbohydrates are found throughout the egg either as free carbohydrates or glycoconjugates (32). The bulk component of egg is the albumen or egg white that constitutes 60% of cumulative egg weight, whereas protein and water add up to other major components (31, 32). The major egg white's proteins are ovalbumin, ovotransferrin, and ovomucoid. Other proteins include ovomacroglobulin (ovostatin), cystatin, lysozyme, avidin, ovoinhibitor andovomucin that gives the albumen its characteristic viscosity (32).

### Dietary Benefits of Eggs

Eggs are rich in complete proteins that promote muscle protein synthesis and maintenance of skeletal mass (34, 35). On average, one large egg provides ~6.3 g protein that is rich in essential amino acids (36). Eggs are affordable nutritious dietary components with significant health benefits (37). These nutrients include vitamins, essential proteins, minerals, fats, and various bioactive compounds. Eggs contain high nutrients to energy density ratio per egg, while also providing numerous essential nutrients (38). For instance, a typical boiled egg weighing ~50 g can provide as much as 78 kcal energy, 0.56 g carbohydrate, 6.29 g protein, and 5.3 g total fat. The total fats from an egg contains up to 1.6, 0.7, 2.0, and 186 mg of saturated, polyunsaturated, monounsaturated fats, and cholesterol, respectively. The micronutrients from eggs include iron, calcium, phosphorus, zinc, potassium magnesium, sodium, and most vitamins except vitamin C. These vitamins include riboflavin, niacin, thiamin, folate, and vitamins A, B6, B12, D, E, and K. Besides providing high quality proteins comparable to breast milk, eggs are also a source of antioxidants. Notable antioxidants from eggs include phosvitin rich in phosphoserines, ovotransferrin that chelates Fe^3+^and ovalbumin that improves the polysaccharide's antioxidant activity through covalent binding (39). These antioxidants act by chelating metal ions and scavenging free radicals thereby inhibit the lipids oxidation. Therefore, eggs are potential sources of natural antioxidants that can be used in both cosmetic and food industries. The eggs' antioxidant activity can ameliorate many degenerative conditions, such as cardiovascular diseases (CVD) in humans (40). However, eggs are not only rich in bioactive components and vital nutrients but also in complete proteins and nutritional cholesterol (28, 41). This controversy has resulted in divergent opinions in dietary recommendation of across populations. Nonetheless, owing to their nutritive quality, eggs, and their derivatives are known to regulate inflammation and modulate immunity.

### Eggwhite Proteins: Evidence of Immune System Enhancement

Egg white part is rich in bioactive compounds with antimicrobial activity (42, 43), besides immunoprotective proteins, which include ovalbumin, ovotransferrin, ovomucin, lysozyme, and avidin that account for 54, 12, 3.5, 3.4, and 0.5% of the egg white proteins by weight, respectively (42). These proteins exert antimicrobial and immunomodulatory effect through direct action on inflammatory pathways (44, 45).

Inflammation is a conventional physiological reaction to infection by pathogens and physical tissue disruption, but it has also been associated with chronic metabolic disorders (23, 46). Inflammation can be regulated by both nutritional and bioactive supplements thereby reducing the disease risk or pathogenesis (24, 25).

Eggs are the best example of dietary components with immunomodulatory effects (26). Studies have shown that egg proteins and its derivative peptides act on various immunomodulatory pathways (Table 1). For instance, ovalbumin modified by methylglyoxal stimulates RAW 264.7 macrophages to secrete TNF-α (56). Elsewhere, Rupa et al. (47) reported that heat denatured ovalbumin modulates cytokines production by CD4+ T cells. Heat-denatured ovalbumin also modulates interleukins' (IL) production by downregulating IL-4 and upregulating IL-10, 12, and 17. Ovalbumin-derived peptides corresponding to amino acids in position 77–84 and 126–134 also enhances the phagocytic activity of macrophages (57) and are reported to enhance immunotherapy (50, 53, 58). Ovotransferrin, another egg white protein, has been shown to stimulate the production of IL-6 and metalloproteinase (MMP) in HD 11 chicken macrophages (59). Xie et al. (59) also reported that ovotransferrin stimulates murine macrophages to produce proinflammatory cytokines through the MAPK signaling pathway. Notably, IL-6 is one of the adaptive immunity components. During chronic inflammation, IL-6 activates T cells, enhances the B cells proliferation and upregulates antibody production. The peptides produced when ovomucin is cleaved by alcalase also express anti-inflammatory activity by inhibiting TNF-mediated NF-κB pathway (49). Tanizaki et al. (60) reported that ovomucin glycoproteins can stimulate macrophages through elevated production of IL-1 and hydrogen. Furthermore, egg white's cystatin enhances the nitric oxide (NO) production by IFN-γ-activated macrophages as a result of IL-10 and TNF-α cytokines activity (61). Cystatin also affects gingival fibroblasts' production of IL-6 and 8 cytokines (62). In another study, Sugahara et al. (63) reported lysozyme-mediated production of immunoglobins. Lysozyme-mediated upregulation of IgM synthesis has been shown to be effective in the management of chronic sinusitis bronchitis and sinusitis (55, 64). Elsewhere, Ha et al. (54) reported the immunomodulatory activity of Maillard-type lysozyme-galactomannan conjugate through NO-enhanced cytokines production in macrophages. This immunomodulatory effect also originates from stimulation of ERK, NF-κB, and JNK pathways. Livetin from the egg yolk has been reported to suppress proinflammatory cytokines, such as IL-1β, 6, and 10 and TNF-α in macrophages thereby inhibiting inflammation (52). Immunoglobulins (G1, G2, M, and A) have been shown to possess immunomodulatory properties in the management of bacterial and viral diseases. Yolkin from egg yolk has also been reported to inhibit free radical generation, thereby inhibiting oxidative stress and pro-inflammatory cytokines, like IL-1β, 6, and 10 and TNF-α in macrophages (55), although at high temperatures, the rate of immunoglobulins aggregation is greatly enhanced thereby locking immunoglobulins in aggregates before complete denaturation.

**Table 1:** Egg proteins and peptides with immunomodulatory activity.

**Protein**	**Peptides-making enzymes**	**Biological activity**	**Mechanism of action**	**References**
Ovalbumin	Pepsin, chymotrypsin	Immunomodulation	- Stimulate TNF-secretion - Increase IL-12,IL-17 and IL-10 production and decreased IL-4	(47)
Ovotransferrin	Thermolysin, pepsin	Immunomodulation	- Stimulate IL-6 production - Increase proinflammatory cytokines production	(48)
Ovomucin	Alcalase, pronase, papain	Immunomodulation	- Stimulate macrophages activity	(49)
Cystatin		Immunomodulation	- Induce TNF, IL-10 synthesis	(50)
Lysozyme		Immunomodulation, antimicrobial	- Stimulate immunoglobulinsproduction and improve chronic sinusitis and bronchitis - Increase proinflammatory cytokine production	(51)
Livetin	Pepsin, acalase	Immunomodulation	- Suppress proinflammatory cytokines production	(52)
IgG1	Papain, Peroxidase	Immunomodulationantimicrobial	Inhibit bacterial metabolism by blocking enzymes Stimulate immunoglobulins production Suppress viral and toxin production	(52)
IgG2	Papain, Peroxidase	Immunomodulationantimicrobial	Inhibit bacterial metabolism by blocking enzymes Suppress viral and toxin production Stimulate immunoglobulins production	(52)
IgM	Peroxidase	Immunomodulationantimicrobial	Inhibit bacterial metabolism by blocking enzymes Suppress viral and toxin production	(53)
IgA	Peroxidase	Immunomodulationantimicrobial	Inhibit bacterial metabolism by blocking enzymes Suppress viral and toxin production	(54)
Yolkin	vitellogenin	ImmunomodulationAntioxidative, Neuroprotective	Immunoglobulins production Increase proinflammatory cytokine production Inhibition of free radical generation	(55)

## Fermented Dairy Products as Immune-Enhancing Food

Historically, fermentation has been widely used in food/beverage processing and preservation. Food fermentation improves palatability besides enhancing nutrients' bioavailability. In addition, microbial fermentation can eradicate toxic milk metabolites, such as galactose and lactose, thereby precluding lactose intolerance and galactose accrual (65). This nutritional and health significance results from modulating intestinal microbiome, ultimately enhancing life expectancy. Fermented dairy products have so far been popular in nutritional enhancement and health promotion. For instance, yogurt resulting from synergistic fermentation of lactose in coagulated milk into lactic acid by *Streptococcus thermophilus* and *Lactobacillus bulgaricus* action, led to the production of a plethora of bioactive compounds in fermented milk, while others have not yet been characterized. As a result of fermented dairy foods consumption, live commensal microbiota can enhance the gut's immunological activity and modulate tolerance from foods-derived antigens (66, 67). The synergy between the immune system and gut microbiome is partially understood compared to the bacteria induced by fermentation by-products.

### Nutritional Value of Probiotic Yogurt

First, it is essential to highlight that a probiotic yogurt is not only a rich source of proteins and fats but also contains numerous essential nutrients. As much as 8 g fats and 9 g of proteins can be sourced per yogurt serving which is more than enough of the recommended daily protein intake. Yogurt is also rich in micronutrients, such as phosphorus, zinc, calcium (33% recommended daily intake, 1,000 mg), vitamin A (10% recommended daily intake), vitamin B12 (>40% recommended daily intake), and pantothenic acid and riboflavin as per Canada's health food standards. As a fermented dairy product, yogurt is also potentially effective in alleviating gastrointestinal (GI) complications, such as constipation, lactose intolerance, *Helicobacter pylori* infection and inflammatory bowel diseases (IBDs) (68, 69). Studies have also suggested that human immunity can be enhanced by yogurt since it influences the intestinal microbiota's equilibrium thereby stimulating the GI immunity through lactic acid and other bacterial metabolites (70, 71).

Despite this marvelous nutritional significance, a reduced consumption of fermented dairy products is associated with a weakened immunity and elevated risk of infectious diseases. In the COVID-19 perspective, GI complications have been commonly reported in patients whose clinical conditions deteriorates rapidly (72). However, administration of gastric medications need to be approached cautiously on case-by-case basis considering their potential to disrupt the immune response thus interfering with COVID-19 treatments currently under evaluation (73, 74). However, the suggested probiotics' health effects from fermented dietary products can be realized only when cell viability is reliable (75). So, to ensure probiotics survival through both GI tract and food processing/storage, the minimal recommended presentation of bacteria is 10^6^-10^7^ colony-forming units (CFUs)/mL organism in each food product (76), despite other factors should also be considered when adding probiotic bacteria in fermented food products to ensure the bacteria's survival. Among them, the compatibility of fermented dairy foods with a wide range of probiotics needs their consideration as “the most efficient delivery vehicle” for probiotics (77, 78), while supplementing fermented milk with probiotics represents an exemplary mode of replenishing the gut microbiota (79).

### The Role of Probiotics in Enhancing the Immune System

The healthy benefits from fermented foods including yogurt have been recognized for a long time (80). Regular consumption of yogurt replenishes beneficial bacteria and macronutrients that enhance immunity by specifically boosting cell immunity, while downregulating oxidative stress (81, 82). Yogurt fortification with probiotics has been shown to greatly enhance the beneficial effects from a health perspective (83, 84). Typically, probiotic bacteria can survive to extreme conditions in the GI tract and maintain their functional benefits where other bacterial strains are catabolized (85). There are many probiotic microbes each with unique biological features, including therapeutic significance. For instance, some probiotic strains are effective in enhancing GI regularity and improving lactose tolerance, whereas others beyond this review's scope have been shown to prevent acute upper respiratory tract infections and bacterial vaginosis. Probiotics within the GI tract inhibit both pathogenic microbes adherence and proliferation (83, 86). This is important in immune homeostasis, since probiotic supplements rejuvenate immunity by balancing the gut microbiome, ultimately stabilizing TREG and Th17 cells (87, 88). Moreover, it can be hypothesized that probiotics consumption can potentially dampen the cytokine storm in combination with appropriate antiviral regimens. Despite the gut microbiota's susceptibility to viral perturbation, it can be equilibrated by dietary interventions. Specifically, to what concerns to COVID-19, gastroenteritis and respiratory distress are some of the most common symptoms (89). By hosting the bulk of body's lymphoid tissues, the GI tract largely influences the immune function when its integrity is altered (90). To this effect, both pre and probiotics able to equilibrate GI microbiota have been reported to reduce the risk of infection (91).

Probiotics have been shown to neutralize inflammation by inhibiting NK cells activation and equilibrating the GI barrier and gut microbiome. However, the exact mechanisms through which probiotics modulate immunity are not perfectly understood (92, 93). *Lactobacillus casei* Shirota (LcS) is a widely studied probiotic with immunomodulatory effects (94), being able to enhance immune system against complications from post-operative infection (95), cancer (96), allergies (97), and to regulate GI functions (98). This probiotic boosts the beneficial gut's bacteria species, augments NK cell activity while maintaining a proper balance between harmful and beneficial bacteria (99, 100). Furthermore, experimental evidence has shown that LcS promotes IL-12 production by peripheral blood mononuclear cells (PBMC) from healthy humans and enhance NK cells' activity, while heat-inactivated LcS boost the cytokines production, including IL-10, 12, IFN-γ, and TNF-α that enhances CD69 expression in NK cells (94).

Though the synergy between microbiome, fermentation by-products and immune system is not fully known, antigen presentation by dendritic cells is thought to be a key player in this aspect. Thus, dendritic cells are significant immunomodulators by initiating diverse responses to various immune stimulations (101). Dendritic cells from monocytes lineage can also be modulated when they interact with lactic acid bacteria (LAB). This response characterizes infection by bacteria and cell debris. Furthermore, through action of GI fermentative bacteria, other bioactive metabolites with immunomodulatory properties are produced. For example, bioactive products generated by *Bifidobacterium breve* can modulate cytokines production by intestinal epithelial cells. Indeed, *B. breve* triggers milk serum fermentation and forms a supernatant that stimulates both dendritic cells development and activation (101).

### The Role of Prebiotic in Enhancing the Immune System

Prebiotic is another additive on fermented dairy products with immune enhancing potential. Prebiotics are non-digestible food supplements that potentially stimulate development of gut domiciled bacteria with host's health benefits (102). For example, the yeast cell walls metabolism generates mannan oligosaccharide (MOS) that contains 30% glucan, 30% mannan, and 12.5% mannoproteins (103, 104). The MOS ability to bind lectin on pathogenic microbes, such as *Salmonella* spp and *Escherichia coli*, minimizes their intestinal proliferation (105, 106).

Another prebiotic yielded as a by-product of fungal/yeast cell wall catabolism is β-glucan, that derives from D-glucose monomers, and are formed via 1–3 β-glycosidic bonds, while the long side chains have a 1–6 glycosidic bond. β-glucans stimulate sentinel cells to produce cytokines and induce lymphocyte multiplication (107). The three types of lymphocytes include NK, T, and B cells. NK and T cells are involved in innate and adaptive immune responses, respectively, whereas B cells are mainly involved in immunoglobulins production. β-glucan can exert its immunomodulatory potential on three lymphocyte categories.

Fructan is another prebiotic derived from plant polysaccharides hydrolyzation or as a by-product of microbial catabolism of plant polysaccharides. The three classes of fructans include levan, inulin and branched groups. Fructan's biological significance is attributed to its β-glycosidic bond that withstands catabolic activities of digestive enzymes whilst augmenting the levels of commensal bacteria including *Lactobacilli* and *Bifidobacteria* spp., whereas pathogenic microbes such as *E. coli* and *Clostridium pefringens* are inhibited (41, 108, 109). Other oligosaccharides with prebiotic potency include xylo-oligosaccharides, chitosan, galactoglucomannan, and galacto-oligosaccharides, with studies revealing that they have immunomodulatory activity following supplementation with fermented dairy products, acting on both cytokines and immunoglobulins production, and promoting phagocytosis, NK and T cells' activity (110, 111). In conclusion, fortification of fermented dairy products with pre- and probiotics could be beneficial in boosting immune system against infections, possibly including SARS-CoV-2.

### Experimental Evidences

A study utilizing fecal samples from Polymerase Chain Reaction (PCR) confirmed and hospitalized COVID-19 patients demonstrated presence of more opportunistic pathogens while the commensal microbes in the gut were depleted compared to healthy individuals. The samples from healthy individuals had prevalence of *Eubacterium, Roseburia, Lachnospiraceae, Facecalibacterium prausnitzii*. Currently, three registered clinical trials are investigating the effect of pre and probiotics on COVID-19 patients. One of the studies is evaluating the beneficial effects of *Lactobacillus coryniformis* on COVID-19 incidence amongst health care workers with a high risk of SARS-CoV2 (NCT 04366180). Another registered clinical trial (NCT04368351) is focusing on bacteriotherapy effects in the treatment of patients with acute diarrhea, and in preventing intensive care in COVID-19 patients. Lastly, the third clinical trial (NCT04366089) is evaluating the adjuvant uses of oxygen-ozone therapy along with probiotic supplementation in COVID-19 patients. Based on these ideas, both pre and probiotics could be proposed as potential tools to be included in the nutritional treatment of COVID-19 patients. Some of these potentially therapeutic probiotics include *Lactobacillus rhamnosus* and *Bifidobacterium lactis*, that exhibits anti-inflammatory effects while also rejuvenating both innate and adaptive immunity.

## Cheese

Cheese is a milk-derived product, rich in nutrients, such as fats, proteins, essential minerals and vitamins, processed by souring milk or clotting it with renin (112). Cheese processing leads to the formation of a versatile food product with a variety of flavors, textures, and nutritional benefits. Physical features of cheese are influenced by casein content, type, quantity, and their interactional strength, its proximate composition and ripening forms (113).

### Nutritional Value of Cheese

Cheese has high nutritive value, being rich in proteins, fat, vitamins, and minerals like calcium (Ca^2+^) and phosphorous (114). Save for cysteine and methionine, cheese is rich in all essential amino acids enough for recommended human intake (115). Besides being significant in human nutrition, proteins are currently considered as important sources of bioactive peptides. These are amino acid sequences that give to a modified protein its characteristic biological activity. These bioactive peptides exert their actions through downregulating blood pressure, chelating minerals, exerting antimicrobial effects, modifying immune system, reducing inflammation, and cholesterol levels (116).

### Cheese as an Immunity Enhancing Product

Studies have shown association between low protein levels (especially immunoglobulins) from cheese and an enhanced risk of infection (114). These nutritional supplements are important in regulating inflammation and oxidative stress responses both known to influence immunity (117). The nutritional impact on immune response is a key aspect in the formulation of anti-inflammatory dietary index (117). For example, dietary supplements rich in omega-3 fatty acids are known to have excellent antioxidant and anti-inflammatory effects, and also a good immunomodulatory potency (118). Elsewhere, experimental protein feeding below 0.8 g/kg body weight in mice, predisposed the animals to severe influenza infection, due to insufficient antibody response, persistence lung viremia, and elevated inflammation with fatal outcomes (119), whereas cheese-derived dietary products rich in calories and saturated fats inhibits inflammation and lipogenesis (120). Thus, the dietary incorporation cheese can reduce post-prandial lipogenesis and inflammatory activities [60]. Furthermore, it has been stated that protein meals, rich in essential amino acids can modulate post-meal glycemic stimuli and improve satiety, since they have better gastric retention and prolonged GI transit (121). This underscores the significance of high quality cheese-derived nutrients, such as calories and saturated fats, as anti-inflammatory enhancing diets that can be incorporated in nutritional immune modulation (122).

## Milk

Milk is one of the main dietary sources of protein for humans, and is primarily composed of water (87%), lactose (5%), fats (0.3%), proteins (4%), vitamins (0.1%), and minerals (123). Milk protein can be categorized as either insoluble (casein) or soluble (whey), with latter constituting 20% of milk proteins, while insoluble proteins constitute the remaining fraction (80%) (124). Both protein categories can sufficiently meet the human's amino acid requirements, besides being digestible and bioavailable. In newborns, milk forms the primary source of bioactive molecules, acting on immune system enhancement against bacteria and viruses and promoting the development of both GI system and bones. The antiviral activity of milk has been attributed to bioactive components with immunomodulatory and anti-inflammatory potential, such as casein, whey proteins and associated peptides (41).

### Dietetic Benefits of Milk

Milk is rich in nutritional components essential for a healthy growth and human development. Minerals, such as calcium is vital in regulating high blood pressure besides being crucial in teeth and bones formation, whereas selenium is a key antioxidant that boosts immune system. The active biological compounds present in milk, including essential amino acids and fatty acids makes it superior to meat in terms of biological significance and value. A good example of these fatty acids is omega-3 which has been implicated in inhibiting some types of cancer and CVD. Caseins, mainly classified as alpha, beta and kappa, act by chelating minerals, such as phosphorus and calcium thereby transporting them as a coagulum in the gut (125).

Recent studies have proposed a correlation between beta casein protein components and human health (126, 127). Bovine milk is an ideal protein source containing all essential amino acids, and high levels of branched chain amino acids, thereby considered as a reference baseline for quantifying other proteins with huge nutritive value (3). Branched chain amino acids, particularly leucine, have been indicated in enhancing synthesis of muscle proteins. Whey protein is also rich in methionine and sulfur-containing amino acids, crucial for glutathione formation, to whom great antioxidant and immunomodulatory effects have been attributed, besides being effective against cancer cells (4, 99).

### Casein Proteins

Casein is a heterogenous protein family, mainly composed of calcium-phosphate micelle complexes (97), classified into alpha-, beta-, gamma-, and kappa-casein (128, 129). Casein and its derivative peptides are known to modulate immune responses, ultimately enhancing the antiviral activity while mitigating sepsis by immune downregulation (126, 130). Caseins also activate B and T-cell mediated responses, thereby linking innate to adaptive immunity. Several casein-derived peptides are also known to have human health benefits by stimulating CV, digestive, immune and central nervous (CNS) systems. For example, some peptides have shown good antioxidant (131), cytoprotective, immunomodulatory (126), antithrombotic and anti-hypertensive effects (131). An example of a peptide that interacts with the CNS is β-casomorphine that exert analgesic effects by acting like opioids (132). Studies have also demonstrated peptides' gut interference via enhanced mucin production, thereby inhibiting the pathogens adherence while enhancing the GI retention which has been linked to a better weight management, through regulated food intake (133).

### Whey Proteins

Whey proteins are specific high-energy crude nutrients with various textures and thicknesses. They often accompany other palatable foods provided to hospitalized patients to improve protein-calorie intake. In addition, they have been associated with immunomodulatory benefits besides complementing antiviral regimens in patients under medication (134, 135).

Whey proteins found in milk are soluble heat labile globular structured compounds stabilized by intermolecular disulfide linkages. Being a heterogenous protein, it is composed of polymorphic proteins including beta-lactoglobulin and alpha-lactalbumin that confers the characteristic foaming and gelation properties (124, 136). Proteose, peptone, immunoglobulin, and glycomacropeptide found in whey have also shown *in vitro* and *in vivo* bioactivities (137). Other whey constituents include lactoferrin, bovine serum albumin (BSA), lactoperoxidase, non-protein components, such as vitamins, fats, lactose, and minerals (138, 139). Lactoferrin is an important immunomodulator with interesting antimicrobial and antioxidant effects. Animal and human experiments have even shown that oral lactoferrin administration is effective against infections, cancer and inflammation, thereby making it an appropriate food additive (140).

#### Immune System Enhancement by Whey Proteins

Experimental evidence has demonstrated that whey proteins have huge immune response enhancing properties. For instance, whey was able to elevate mucosal antibody response against cholera toxin and ovalbumin when fed to mice for 12 weeks compared to control on a regular diet (141). In another study, bovine whey proteins consumption (for 5–8 weeks) by mice led to a marked raise on foot pad delayed-type hypersensitivity responses and *in vitro* concanavalin A-induced spleen cell proliferation (141). The helper T cell (L3T4+) populations were also elevated in mice receiving undenatured whey protein (25 g) for 4 weeks compared to the control group fed an isocaloric casein diet (142). Not only the L3T4+ cells population were elevated but also the helper (L3T4+) to suppressor T-cells (Lyt-2+) ratio too. In comparison to casein and soy protein rich diets, whey protein-diet has been linked to an elevated count of CD4+, and C8+ lymphocytes, total white blood cells and increased IFN-γ production by concanavalin A-stimulated spleen in experimental mice (143). Whey proteins also increase the glutathione levels in plasma and enhance the NK cells activity in patients with chronic hepatitis B (144). Moreover, whey proteins' antiviral activity has been reported against HIV. Further, β-LF strongly inhibits reverse transcriptase and mildly inhibits integrase and protease, whereas β-LG and α-LA inhibits integrase and protease but not reverse transcriptase during the early infection stages (145). Elsewhere, whey derivatives, such as β-lactoglobulin and α-lactalbumin have been shown to bind and deter attachment of rotavirus to the host's cells receptors (146). In addition, lactoperoxidase enzyme from whey when combined with thiocyanate and hydrogen peroxide substrates also exhibits antiviral activity against herpes, HIV and echovirus (147, 148). Moreover, when orally administered, lactoperoxidase can mediate pneumonia disruption in mice experimentally infected with influenza by suppressing inflammation in lung cells (149).

## Conclusion

SARS-CoV-2 infection has triggered a serious global devastation in human being's well-being and health maintenance, and a huge impact in healthcare delivery systems. Elderly individuals are particularly vulnerable to such infection, as both physiological and immunological performance declines with age, ultimately raising the risk of refractory infections and gets serious ill. Hence, implementation of infection control programs is difficult for them. This review mostly aimed to clarify the value of systemic dietary distortions in COVID-19 patients, as immunity is impaired by a poor nutritional status. For that purpose, here the different types of food that enhance humans' immune response, such as egg-derived proteins, milk, and fermented dairy products containing different probiotics were addressed and discussed. Eggs, yogurt, cheese, and milk are rich sources of essential macro and micronutrients, that possess excellent immunomodulatory, anti-inflammatory, antioxidant and antiviral activities, being thus essential for the maintenance of a healthy growth and development.

## Author Contributions

GB, DA, AA, RN, MW, MS, MNA, BA, NC-M, NK, HH, AG, and J-MS wrote and carefully revised the paper. All authors have read and agreed to the published version of the manuscript.

## Conflict of Interest

The authors declare that the research was conducted in the absence of any commercial or financial relationships that could be construed as a potential conflict of interest.
